# The cerebral cavernous malformation 3 gene is necessary for senescence induction

**DOI:** 10.1111/acel.12316

**Published:** 2015-02-05

**Authors:** Ana Guerrero, Cristina Iglesias, Selina Raguz, Ebel Floridia, Jesús Gil, Celia M Pombo, Juan Zalvide

**Affiliations:** 1 Department of Physiology, CIMUS, Instituto de Investigación Sanitaria IDIS, University of Santiago de CompostelaSantiago de Compostela, A Coruña, 15703, Spain; 2 Cell Proliferation Group, MRC Clinical Sciences Centre, Imperial College LondonLondon, W12 0NN, UK; 3 Quantitative Cell Biology, MRC Clinical Sciences Centre, Imperial CollegeLondon

**Keywords:** autophagy, CEBPb, PDCD10, SASP

## Abstract

Mutations in cerebral cavernous malformation 3 gene are known to result in development of vascular malformations and have recently been proposed to also give rise to meningiomas. We report in this study that lack of CCM3 unexpectedly impairs the senescence response of cells, and this is related to the inability of CCM3-deficient cells to induce the C/EBPβ transcription factor and implement the senescence-associated secretory phenotype. Induction of C/EBPβ and cytokines is also impaired in the absence of CCM3 in response to cytokines in nonsenescent cells, pointing to it being a primary defect and not secondary to impaired senescence. CCM3-deficient cells also have a defect in autophagy at late passages of culture, and this defect is also not dependent on impaired senescence, as it is evident in immortal cells after nutrient starvation. Further, these two defects may be related, as enforcing autophagy in CCM3-deficient late passage cells increases C/EBPβ cytokine expression. These results broaden our knowledge on the mechanisms by which CCM3 deficiency results in disease and open new avenues of research into both CCM3 and senescence biology.

## Introduction

Cellular senescence develops in response to a variety of stresses, including telomere attrition, unscheduled DNA replication, oxidative stress, suboptimal culture conditions, or the presence of an activated oncogene (in the latter case being called oncogene-induced senescence, OIS) (Hayflick, 1965; Kuilman *et al*., 2008). It was first described in cells in culture and is now widely accepted as an important antioncogenic mechanism *in vivo* (Braig *et al*., 2005; Collado *et al*., 2005; Michaloglou *et al*., 2005).

Some of the best known characteristics of senescent cells are those that are directly related to their growth arrest, such as the activation of p53 or the upregulation of the cyclin-dependent kinase inhibitors p16^ink4a^ or p21^CIP1^ (Campisi & d'Adda di Fagagna, 2007). However, in the last few years, several other physiological changes have been shown to be important for the full implementation of the senescence program; among these are the secretion of a plethora of extracellular messengers, most prominently cytokines, by senescent cells (Krtolica *et al*., 2001; Coppé *et al*., 2006; Acosta *et al*., 2013; Kuilman *et al*., 2010; Kuilman & Peeper, 2009), and the stimulation of the process of macroautophagy (usually called simply autophagy) (Kurz *et al*., 2000; Young *et al*., 2009; Narita *et al*., 2011). Autophagy is necessary in this context for the efficient synthesis and secretion of extracellular messengers (Young *et al*., 2009), and once established, the synthesis and secretion of cytokines can be self-sustained by a positive feedback loop involving the transcription factors NFκB and C/EBPβ (Acosta *et al*., 2013).

Mutations in the CCM3/PDCD10 gene (CCM3 from here on) predispose to cerebral cavernous malformations (CCM, OMIM #116860), a common type of vascular malformation which develop almost exclusively in the central nervous system (Rigamonti *et al*., 1988). Its product is an adaptor protein that binds to the germinal center kinase III (GCKIII) family of protein kinases (composed of Mst3/STK24, Mst4/MASK, and SOK1/YSK1/STK25) through its N-terminal domain (Ma *et al*., 2007; Voss *et al*., 2007; Fidalgo *et al*., 2010; Zalvide *et al*., 2013), and to other proteins through its C-terminal end (Voss *et al*., 2009; Goudreault *et al*., 2008; Fidalgo *et al*., 2010; Li *et al*., 2011). Several functions have been proposed for CCM3, including modulation of cell death, especially after oxidative stress (Chen *et al*., 2009; Schleider *et al*., 2010; Fidalgo *et al*., 2012; Zhang *et al*., 2012), regulation of transmembrane signaling and cell growth (Ma *et al*., 2007; Kleaveland *et al*., 2009; He *et al*., 2010; Lin *et al*., 2010), and playing a role in membrane trafficking, Golgi apparatus biogenesis, cell migration, and regulated secretion (Fidalgo *et al*., 2010; Zhang *et al*., 2013; Louvi *et al*., 2014).

Recently, patients carrying heterozygous CCM3 gene mutations have been shown to be at high risk of developing meningiomas in which the wild-type allele of CCM3 is mutated (Labauge *et al*., 2009; Riant *et al*., 2013), which hints to a possible role of CCM3 as a tumor suppressor, although no mechanism for such an effect has been proposed.

Here, we show that cells deficient in CCM3 do not enter senescence after replicative stress or oncogene induction. Lack of CCM3 results in impaired expression of cytokines and their regulator C/EBPβ, both in senescence and in response to cytokines. Moreover, CCM3-deficient cells do not increase autophagy at late passages of culture or after nutrient starvation.

## Results

**Figure 1:**
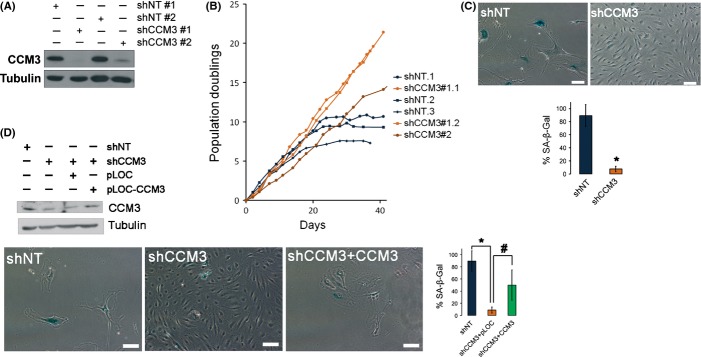
CCM3-deficient endothelial cells do not undergo proliferative arrest. HCAEC cells were lentivirally infected with two different shRNA against CCM3 (shCCM3 #1 or shCCM3 #2) or with a nontarget shRNA (shNT #1 or shNT #2). (A) CCM3 knockdown efficiency was assessed by Western blot. Tubulin is shown as a loading control. (B) Cumulative population doublings in six independent populations of primary HCAEC cells transduced either with a nontarget shRNA (shNT), shCCM3#1, or shCCM3#2. (C) Microphotographs showing SA-βGal activity in HCAEC shNT cells at population doublings 18, and shCCM3#1 cells cultures in parallel. Representative photographs and a quantification of SA-βGal-positive cells (mean ± SEM), *n* = 3. *P = 3.07 × 10^−6^. (D) Western blot of CCM3 showing the recovery of its levels after transduction with pLOC or pLOC-CCM3 (upper panel); representative photographs of SA-βGal activity in HCAEC shNT and shCCM3#1 cells transduced with pLOC or pLOC-CCM3 (lower panels); and quantification of SA-βGal-positive cells (mean ± SEM), *n* = 3. *P = 2.4 × 10^−6^, #*P* = 0.018.

To study the biology of the cavernous malformation susceptibility gene CCM3, we depleted the CCM3 protein in primary endothelial cells by lentiviral transduction of CCM3 small hairpin RNAs. Two of the shRNAs – shCCM3#1 and shCCM3#2 – we used gave a good downregulation of CCM3 (Fig.1A). As expected for primary cells, those infected with a control shRNA stopped their proliferation when they reached between 6 and 12 population doublings, and showed the typical morphology of senescent cells. Surprisingly, cells with a downregulated CCM3 continued to divide when they reached the same number of doublings. Cells infected with shCCM3#1 did not show a clear slowing in their proliferation rate even after 25 population doublings, whereas cells infected with the less effective shCCM3#2 shRNA had an intermediate phenotype, proliferating longer than the control cells but ultimately ceasing their division (Fig.1B). Significantly, the proliferation during the first divisions of the cells was undistinguishable in cells with different levels of CCM3 (duplication time of 52.4 ± 11.9 h for control vs. 56.7 ± 14.2 h for CCM3 knockdown cells, *P* = 0.47), and their cell cycle profile was also similar (Fig. S1A, Supporting information), which pointed to a specific senescent defect. In the same analysis, no difference in sub-2n DNA was found, suggesting also that lack of CCM3 does not induce apoptosis at least in unstressed endothelial cells.

Consistently with an involvement in senescence, CCM3-depleted cells did not acquire the morphology characteristic of senescent cells even after 25 population doublings, and they did not accumulate senescence-associated β-galactosidase (Fig.1C). CCM3-depleted cells were clonogenic, but did not form foci when allowed to grow to confluence (Fig. S1B,C, Supporting information), and did not accumulate the cdk inhibitors p21^cip1^ or p16^ink4a^ (Fig. S1D,E, Supporting information). They did show signs of low-intensity DNA damage, as seen by γH2AX levels (Fig. S1F, Supporting information), and higher p53 levels than low passage cells, although p53 could still respond to the intense DNA damage induced by doxorubicin (Fig. S1G, Supporting information). To further establish whether the effects of the shRNAs were due to CCM3 depletion and not to nonspecific effects of the shRNAs, we reexpressed CCM3 with retroviral transduction. The shCCM3 #1 shRNA targets the 3′ UTR of the CCM3 mRNA, and therefore, it does not have any effect on a construct with the coding region of CCM3. CCM3 transduction could recover CCM3 protein levels partially, and this was enough to increase significantly the number of positive cells for SA-βGal activity (Fig.1D), indicating that senescence was being rescued.

To know how widespread the relation of CCM3 to senescence was, we depleted this gene from IMR90 fibroblasts using a CCM3 shRNA unrelated to those previously used (Fig. S2A, Supporting information). CCM3-depleted fibroblasts did not enter senescence at the same passage as control cells (Fig. S2B, Supporting information), although they showed signs of senescence three passages later (not shown), suggesting CCM3 only delays senescence in this model.

We hypothesized that if CCM3 was specifically required for replicative senescence, its expression might be increased during the process. However, CCM3 mRNA levels did not vary significantly as IMR90 cells accumulated population doublings (Fig. S2C, Supporting information), suggesting that if CCM3 is activated, it is through a mechanism different than mRNA expression.

**Figure 2:**
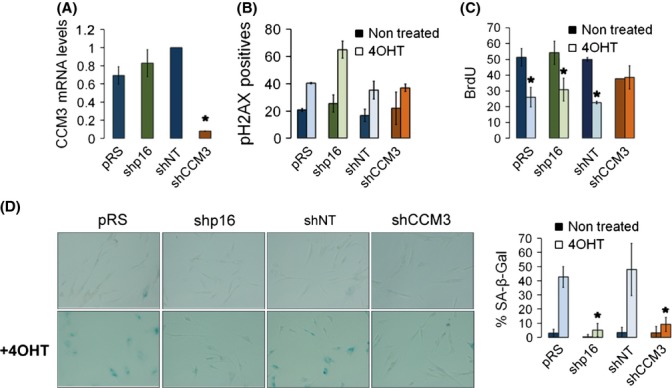
CCM3 depletion bypasses oncogene-induced senescence (OIS) in IMR90 cells. IMR90 cells expressing a switchable version of H-Ras (activated upon addition of 4-hydroxytamoxifen, 4-OHT) were infected with a lentiviral CCM3 shRNAs (shCCM3) or nontarget shRNA (shNT), or with a retroviral p16 shRNA (shp16) or nontarget shRNA (pRS), and then selected with puromycin. (A) CCM3 mRNA levels were assessed by qRT–PCR. *n* = 3, *P = 0.012 vs. shNT. (B) pH2AX-positive cells in the different populations untreated or after 5 days of H-Ras activation (4-OHT). For B and C, the nuclear intensity average of the staining correlated to the shown percentage of positive cells. (C) Percentage of BrdU-positive cells treated as in B, *n* = 3. *0.002, 0.005, 0.0002 vs. untreated control. (D) SA-βGal activity in the same cells under the same treatments as in B. Shown are representative photographs and a quantification of SA-βGal-positive cells by two independent observers. *P = 0.0003 and 0.0002 vs. pRS cells treated with 4-OHT.

We then studied the effect of CCM3 in senescence in a well-known model of senescence induction: IMR90 cells transduced with an oncogenic H-ras switchable with the estrogen analog 4-hydroxytamoxifen (4-OHT). When CCM3 was inhibited in these cells (Fig.2A), 4-OHT could still increase the number of γH2AX-positive cells (Fig.2B), showing that H-ras was active in both control and CCM3-deficient cells. However, it did not inhibit DNA synthesis (Fig.2C), nor did it induce a senescent morphology or senescence-associated β-galactosidase activity (Fig.2D). Moreover, depletion of CCM3 with completely unrelated shRNAs transduced by retroviral vectors had the same effect (Fig. S2, Supporting information), showing again that it is CCM3 downregulation and not a nonspecific effect of CCM3 shRNAs what induces the senescence bypass.

**Figure 3:**
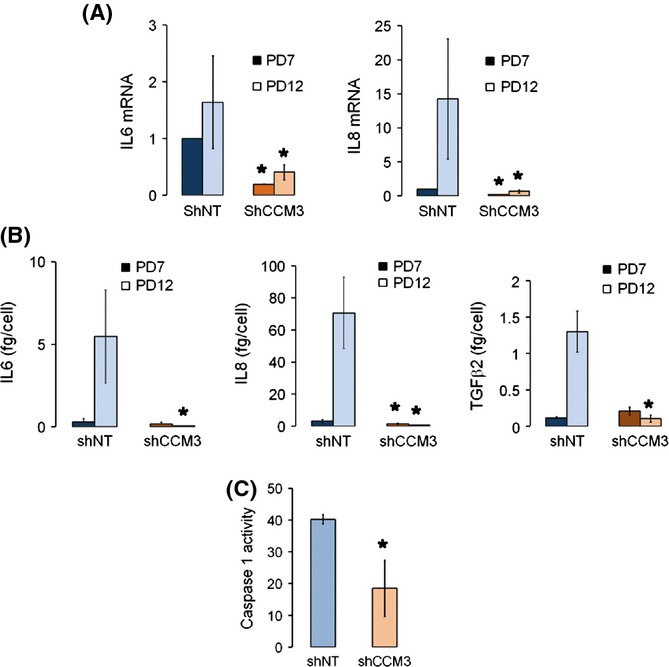
CCM3 knockdown endothelial cells show lower levels of SASP components and inflammasome activation. (A) mRNA levels of IL-6 and IL-8 from shCCM3 and shNT cells with 7 (PD7) or 12 (PD12) population doublings. *n* = 4, *P = 0.019, 0.023; and 0.017, and 0.04 vs. shNT. (B) Secretion of SASP components (IL-6, IL-8 and TGF-β2) in cell culture supernatants. *n* = 3, *P = 0.028, 0.048, 0.035, and 1.25 × 10^−4^ vs. shNT. (C) Activity of caspase-1 in late passage (PD18) shNT and shCCM3 HCAEC cells. *P = 1.8 × 10^−4^.

Once we concluded that CCM3 depletion inhibited senescence in several different models, we wanted to understand how CCM3 could affect senescence in our original endothelial cells. Thus, we performed a transcriptomic analysis comparing expression of genes regulated in senescence in control cells (‘senescence genes’) between passage 12 shNT and shCCM3 cells (Fig. S4A, Supporting information). Gene set enrichment analysis of this comparison against KEGG datasets showed that two sets related to senescence were downregulated in shCCM3 cells: cytokine–cytokine receptor interaction and lysosome (Fig. S4B, Supporting information). Indeed, expression of IL-6 and IL-8 mRNAs was lower in late passage CCM3-depleted cells than in control cells as assessed by quantitative RT–PCR (Fig.3A). Also, while late passage control cells secreted high quantities of IL-6, IL-8, and TGF-β2 to their medium, cells without CCM3 did not (Fig.3B). Interestingly, control cells at high passage had a higher activity of caspase-1 than shCCM3 cells (Fig.3C), suggesting that the inflammasome is activated differently depending on the CCM3 status, which might affect cytokine response to inflammation-related stimuli.

**Figure 4:**
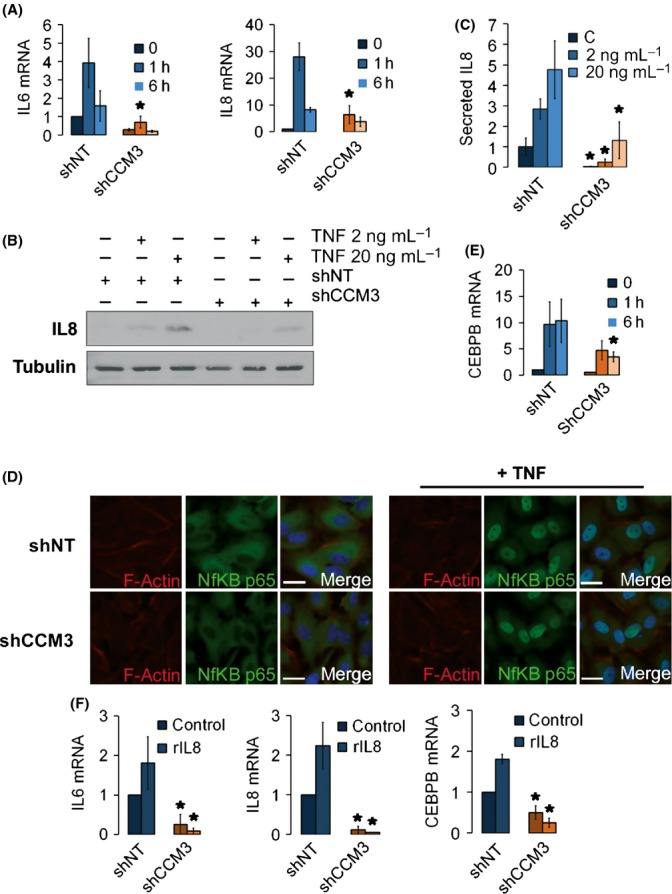
CCM3 is necessary for cytokine-induced C/EBPβ and cytokine induction. (A) Induction of IL-6 and IL-8 mRNAs by TNF is dependent on CCM3. Control (shNT) and shCCM3 HCAEC cells were either treated for 1 or 6 h with TNF 20 ng mL^−1^, or left untreated. Levels of IL-6 and IL-8 mRNAs were determined. *n* = 3, *P = 0.028 and 0.003 vs. shNT cells. (B) IL-8 induction by TNF depends on CCM3. HCAEC cells were either treated with 2 ng mL^−1^ or 20 ng mL^−1^ TNF, or left untreated. IL-8 levels were determined by Western blot. Tubulin is shown as a loading control. (C) Stimulation of IL-8 secretion by TNF depends on CCM3. Levels of IL-8 were determined in cell culture supernatants by ELISA, after treatment with 2 ng mL^−1^ or 20 ng mL^−1^ TNF. *n* = 3, *P = 5.0 × 10^−3^, 2.4 × 10^−4^, 7.3 × 10^−3^ vs. shNT. (D) CCM3 status does not affect NFκB nuclear translocation. Immunofluorescence showing NFκB activation after TNF 20 ng mL^−1^ treatment in control and shCCM3 cells. The bar represents 20 μm. (E) TNF induces C/EBPβ mRNA in a CCM3-dependent manner. HCAEC cells were either treated for 1 or 6 h with TNF 20 ng mL^−1^, or left untreated. Levels of C/EBPβ mRNA were determined. *n* = 3, *P* = 0.035 vs. shNT cells. (F) Induction of cytokines and C/EBPβ by IL-8 is dependent on CCM3. Control (shNT) and shCCM3 HCAEC cells were grown in the presence or absence of 200 ng μL^−1^ h^−1^ IL-8, and levels of IL-6, IL-8 and C/EBPβ mRNAs were determined. *n* = 4, *P = 0.004, 0.007, 0.0001, 0.001, 0.035, and 0.023 vs. shNT.

Regulation of cytokines during senescence depends on a network of positive feedback loops whereby some cytokines stimulate their own expression or that of related molecules (Acosta *et al*., 2013; Kuilman *et al*., 2010; Acosta *et al*., 2008). To assess the regulation of cytokine expression by CCM3, we studied the response of cells to TNF, which is known to regulate IL-8 and IL-6 in endothelial cells. TNF could induce both IL-6 and IL-8 mRNA in a time-dependent manner in control cells, and this response was clearly impaired in CCM3-deficient cells (Fig.4A). The same was true for IL-8 intracellular protein levels and extracellular secretion (Fig.4B,C). Furthermore, TNF could induce the nuclear translocation of the transcriptional cytokine regulator NFκB and increase the mRNA levels of the other principal regulator, C/EBPβ. In the absence of CCM3, the nuclear translocation of NFκB was unaffected while the increase in C/EBPβ mRNA was impaired (Fig.4D,E). This defect in cytokine regulation was not limited to the response to TNF. Treatment with recombinant IL-8 induced the mRNAs of IL-6 and IL-8 in control but not CCM3-deficient cells, and this was also accompanied by an impaired induction of C/EBPβ in the latter (Fig.4F). We concluded that the regulatory network of cytokine regulation was impaired in CCM3-deficient cells, and this was closely related to their inability to induce the C/EBPβ transcriptional regulator.

**Figure 5:**
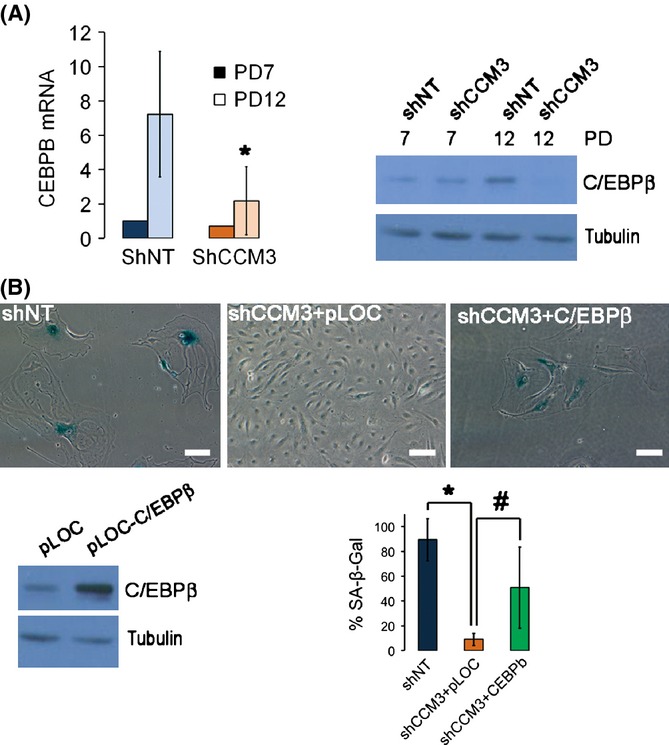
CCM3 is necessary for C/EBPb in senescence and C/EBPb overexpression rescues senescence in CCM3-deficient cells. (A) Induction of C/EBPb in senescence depends on CCM3. C/EBPβ mRNA (graph) and protein (right panel) were determined in control (shNT) and shCCM3 HCAEC cells of population doubling 7 (PD7) or 12 (PD12) by qRT–PCR or Western blot. *n* = 4, *P = 0.029 vs. shNT. (B) Rescue of C/EBPβ induces senescence in late passage shCCM3 HCAEC cells. Lower panel, left: Western blot of C/EBPb in shNT and shCCM3 cells transduced with pLOC or pLOC-C/EBPβ at population doublings 18. Upper panels: Microphotographs showing SA-βGal activity in HCAEC shNT cells at population doublings 18, and shCCM3 HCAEC cells transduced with pLOC or pLOC-C/EBPβ. Lower panel, right: quantification of SA-βGal-positive cells (mean ± SEM), *n* = 3. *P = 2.4 × 10^−6^. #*P* = 0.033.

C/EBPβ was also induced in late passage (senescent) control cells but not in CCM3-deficient cells (Fig.5A). Moreover, when the expression of C/EBPβ was enforced in late passage shCCM3 HCAEC cells, a significant percentage of them accumulated SA-βGal activity and acquired a senescent-like morphology (Fig.5B), suggesting that CCM3 facilitates senescence at least in part through its effects on C/EBPβ expression.

**Figure 6:**
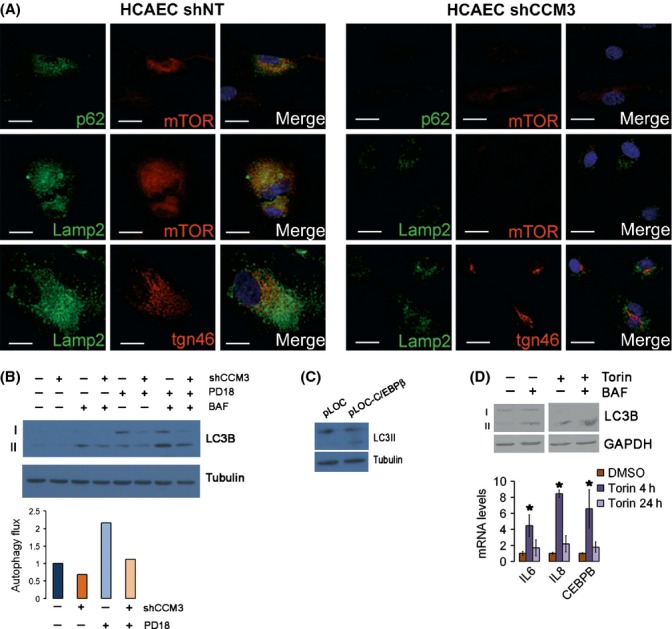
CCM3 is important for autophagy induction during senescence. (A) TOR-autophagy spatial coupling compartment (TASCC) markers in control (shNT) and shCCM3 HCAEC cells as seen by immunofluorescence. Upper photographs show the costaining of p62/SQSTM1 and mTOR. Medium photographs show the costaining of the lysosomal marker LAMP2 and mTOR. Lower photographs show the staining of the trans-Golgi marker TGN46 and the lysosomal marker LAMP2. The bar represents 20 μm. (B) Western blot for LC3B in shNT and shCCM3 HCAEC cells at population doubling 7 and 18. Where indicated, cells were treated with autophagy inhibitor bafilomycin 100 nm for 1 h. LC3B-II levels were determined by fluorescent Western blot. Tubulin is shown as a loading control. Right panel: autophagic flux under each condition. (C) C/EBPβ expression induces autophagy in late passage CCM3-deficient cells. Western blot of LC3B-II in HCAEC cells transduced with pLOC or pLOC-C/EBPβ. (D) Induction of autophagy by mTOR inhibition upregulates IL-6, IL-8, and C/EBPβ levels in endothelial cells deficient for CCM3. Cells were treated with Torin1 for 4 h and incubated with bafilomycin 1 for the last hour where indicated. Accumulation of LC3B-II was quantified by Western blot. Levels of IL-6, IL-8, and C/EBPβ mRNAs were then determined by qRT–PCR after treatment with Torin1 250 nm for the indicated hours. *n* = 3, *P = 0.043, 5 × 10^−6^, and 0.044 vs. untreated cells.

Recently, the combination of autophagy and mTOR activity in a newly defined cellular compartment called TOR-autophagy spatial coupling compartment (TASCC), where lysosomes and mTOR accumulate, has been proposed to be essential for cytokine secretion during senescence. The lysosome gene set is also downregulated in late passage shCCM3 cells, and CCM3 has been implicated in Golgi biogenesis, vesicular trafficking, and regulated secretion (Fidalgo *et al*., 2010; Kean *et al*., 2011; Zhang *et al*., 2013). Thus, we hypothesized that formation of TASCC might be altered in CCM3-deficient cells. As expected, senescent control cells displayed a prominent protein degradation machinery as seen by LAMP2 and p62 staining together with high levels of mTOR, suggestive of the existence of the TASCC complex. On the contrary, cells without CCM3 did not show any signs of TASCC (Fig.6A).

TOR-autophagy spatial coupling compartment is formed by the accumulation of mTOR and lysosomes that result from the high autophagic activity of senescent cells (Narita *et al*., 2011). As CCM3 is involved in vesicular traffic, we reasoned that it might have an effect on autophagy. Thus, we monitored autophagy in early and late passage cells by measuring the lipidated form of LC3B, LC3B-II, by Western blot, and estimated autophagy flux as the difference in LC3B-II levels between cells with and without treatment with bafilomycin A (BAF), which inhibits autophagosome–lysosome fusion (Klionsky *et al*., 2012). As described, autophagy is elevated in late passage control cells. However, late passage cells with silenced CCM3 did not show the same induction of autophagy (Fig.6B).

Our results showed that CCM3-deficient cells had a defect in both autophagy and C/EBPβ induction in several circumstances. Both effects were related. The expression of C/EBPβ in late passage shCCM3 cells induced an accumulation of LC3B-II (Fig.6C). Further, enforced induction of autophagy transiently induced C/EBPβ and cytokine mRNAs. As seen in Fig.6D, the mTOR inhibitor Torin1 could induce autophagy in CCM3-deficient endothelial cells, as assessed by the accumulation of LC3B-II after treatment with bafilomycin. When autophagy was induced, there was an accumulation of IL-6, IL-8, and C/EBPβ mRNAs in CCM3-deficient cells (Fig.6D, lower graph). This induction was visible only at early times after Torin1 treatment and was reverted as soon as after 24 h, which we interpret to reflect the need of mTOR activity in the full induction of senescence.

We reasoned that if autophagy impairment is a primary effect of lack of CCM3, it should be evident independently of senescence. Thus, we inhibited CCM3 in an immortal cell line derived from retinal epithelial cells (hTERT-RPE1) (Fig. S5A, Supporting information) and then induced autophagy by incubating them in Hank's Buffered Saline Solution (HBSS), a medium poor in amino acids. Lack of CCM3 did not apparently affect autophagy flux of hTERT-RPE1 cells when grown in complete medium, but inhibited its stimulation by nutrient starvation (Fig. S5B, Supporting information). When we monitored autophagy by counting the LC3B-positive punctae per cell, we also found that nutrient starvation induced significantly more punctae when CCM3 was present (Fig. S5C, Supporting information). CCM3 did not affect the number of punctae in cells in complete medium significantly, although in this case there was a trend toward there being less punctae in CCM3-deficient cells. Further, impairment of autophagy is a consequence of CCM3 silencing, because enforced expression of CCM3 protein results in recovery of autophagic flux after nutrient starvation (Fig. S5D, Supporting information). We concluded from the above experiments that lack of CCM3 impaired the entry of cells into senescence through effects on autophagy, which resulted in inhibition of C/EBPβ induction, and of the senescence-associated secretome.

## Discussion

We show here that CCM3 is important for senescence in primary cells and propose that the link between CCM3 and senescence is its involvement in autophagy and TASCC formation, which are essential for the induction of the transcription factor C/EBPβ and the production of senescence-associated cytokines. These results shed new light in the actions of CCM3 at the cellular level and into the relations between autophagy, C/EBPβ, and senescence.

CCM3 is involved in autophagy, not only during senescence but also after nutrient deprivation in postsenescent cells. As CCM3 is important for certain aspects of membrane handling in the cell, such as Golgi biogenesis and regulated secretion, we expect it to be involved in the early stages of autophagosome formation. In fact, the ability of CCM3 to bind to phosphatidylinositols on one end and kinases of the GCKIII family on the other (Sugden *et al*., 2013; Zalvide *et al*., 2013), and its action modulating the binding of the STK24 kinase to the secretory regulator UNC13D (Zhang *et al*., 2013), may be important for its autophagy promoting function.

CCM3 is also important in the induction of the transcriptional regulator C/EBPβ, both during senescence and in response to cytokines, two responses that may be related given the importance of cytokine–cytokine networks in the senescence process. The experiments also suggest that C/EBPβ expression depends upon autophagy induction, at least in some settings. Because C/EBPβ can also stimulate autophagy, as it has also been found in other systems (Ma *et al*., 2011; Guo *et al*., 2013), we propose there is a positive feedback loop between this transcription factor and autophagy during senescence. C/EBPβ has been proposed to contribute to the cell cycle arrest in senescence by inducing the expression of cdk inhibitors such as p15ink4b in models where this is the principal inhibitor induced (Kuilman *et al*., 2010). Also, the senescence-associated secretion has been shown to induce many of the features of senescence in a paracrine manner (Acosta *et al*., 2008). Thus, we hypothesize that lack of C/EBPβ expression underlies the lack of induction of senescence markers in CCM3 silenced cells and their inability to growth arrest. This is supported by the rescue of the senescence phenotype by enforced expression of C/EBPβ.

Our results add to our knowledge of cellular functions of CCM3, which has been implicated in cell death and in regulation of cell proliferation. CCM3 overexpression has been shown to induce apoptosis, and its inhibition to protect from necrosis after oxidative stress; and from apoptosis after serum deprivation, cycloheximide treatment, or depletion of γ-protocadherins (Chen *et al*., 2009; Lin *et al*., 2010; Louvi *et al*., 2011; Fidalgo *et al*., 2012). We have not challenged our cells with death inducing factors so that we do not observe cell death which CCM3 might inhibit, either by sub-2n DNA or nuclear morphology. Thus, we propose that the senescent effect of CCM3 is independent of its apoptosis regulating functions. We also do not see an effect on proliferation in early passage endothelial cells. This is consistent with the results reported for HuVEC cells, in which a marginal effect or no effect at all of CCM3 depletion on proliferation is seen (Schleider *et al*., 2010; Zhu *et al*., 2010), and opposed to the clear effect of CCM3 inhibition on proliferation in astrocytes (Louvi *et al*., 2011).

Patients with a heterozygous mutation of CCM3 have a high susceptibility to develop cerebral cavernous malformations, and it has been shown recently that they can also develop meningiomas, which places CCM3 as a possible tumor suppressor gene (Clark *et al*., 2013; Riant *et al*., 2013). Despite intensive research in the last years, the mechanism by which lack of CCM3 (or lack of CCM1 or CCM2) in endothelial cells results in cavernous malformations is still the subject of debate. Defects in cellular death, polarization, migration, adhesion, and also in endothelial barrier functions, angiogenesis, and differentiation have all been proposed as contributing to cavernoma development (for a review, see Fischer *et al*., 2013). Our results add a new defect of endothelial cells lacking a CCM gene, the inability to enter senescence. While endothelial cell senescence is usually related to aging, senescent cells can develop prematurely as a consequence of cell stress, and new evidence suggests that senescence may also be a developmentally regulated process that contributes to the disposal of surplus cells (Munoz-Espin *et al*., 2013; Storer *et al*., 2013). Experiments designed to analyze whether senescent endothelial cells appear in the brain under specific circumstances, such as angiogenesis, are needed to further study the possible relation between senescence and the development of cavernous malformations.

Mutations of the CCM3 gene can also result in multiple meningiomas, and the ability of CCM3 to induce senescence is likely to contribute to their development. This opens a new avenue of research on the relation between CCM3, senescence, and meningioma development.

## Experimental procedures

### Antibodies and plasmids

The antibodies used in this study were as follows: TGN46 (Abcam; ab16052); CCM3 (Acris; AP26023PU-N); BrdU (BD Biosciences; 555627), Lamp-2 (BD Biosciences; 555803), p21^CIP1^ (F-5) (Santa Cruz; sc-6246), p16^INK4^ (BD Biosciences; 511325), p62 (BD Biosciences; 610832); GAPDH (Calbiochem; CB-1001); LC3B (Cell Signaling; 3868), mTOR (Cell Signaling; 2983); H2AX (Ser139) (Millipore; 05-636); IL-8 (500-P28) (PeproTech; 500-P28); NFκB p65 (Santa Cruz; sc-8008); p53 (DO-1) (Santa Cruz; sc-126); C/EBPβ (Santa Cruz; sc-150); and tubulin (T5168) (Sigma-Aldrich). The secondary antibodies used were as follows: goat anti-rabbit DyLight™ 800, goat anti-mouse DyLight™ 680 (Thermo Scientific); goat anti-mouse Alexa 488, goat anti-rabbit Alexa 488, goat anti-mouse Alexa 594, and goat anti-rabbit Alexa 546 (Molecular Probes).

All plasmids were constructed using standard molecular biology techniques.

### Cell culture

HCAEC cells, hTERT-RPE1, and IMR90 cells were obtained from European Collection of Cell Cultures and were grown as recommended. Replicative senescence was induced in HCAEC cells by passaging them every 2 days in MesoEndo Cell Growth Medium 212-500 (Cell Applications, Inc.). Oncogene-induced senescence was induced in ER:Ras-IMR90 by treatment with hydroxytamoxifen for 5 days. All experiments where population doublings are specified refer to shCCM3 cells of those population doublings or shNT cells cultured in parallel for the same time.

### shRNA-mediated knockdown and recovery

Stable cell populations with silenced CCM3 or control were obtained via selection after lentiviral transduction using MISSION lentiviral nontarget shRNA control transduction particles or MISSION lentiviral shRNA transduction particles against human CCM3, from Sigma-Aldrich (TRC), or by retroviral transduction where stated. shRNAs sequences are available upon request. Lentiviral transduction of CCM3 and C/EBPβ was performed using plasmids from the Precision LentiORF Collection (Thermo Scientific Open Biosystems). Transduced cells were then selected by blasticidin.

### Treatments

HCAEC cells were treated with doxorubicin (Sigma-Aldrich) 1 μm for 24 h. hTERT-RPE1 cells were treated HBSS (Invitrogen) for 4 h to trigger autophagy. In the last hour, cells were treated with bafilomycin A1 (Calbiochem) 0.1 μm, or left untreated. HCAEC cells were grown in the presence of recombinant human IL-8 or TNF (R & D Systems) 200 ng μL^−1^. mTOR activity was inhibited with Torin1 (Tocris) 250 nm. IMR90 cells were treated with 4-hydroxytamoxifen (Sigma-Aldrich) 200 nm to activate Ras in IMR90 Ras:ER.

### Immunofluorescence and image analysis

HCAEC and hTERT-RPE1 cells were fixed, permeabilized, blocked, and incubated with primary antibodies overnight at 4 °C in PBS + 1% BSA. DNA was stained with Hoechst 33342. Confocal images were collected using a Leica confocal microscope equipped with an HCX PL APO CS 63x/1.32 objective. Leica LCS software was used for acquisition and analysis. Images are combinations of optical sections taken in the *z*-axis at 0.13-μm intervals. Immunofluorescences in IMR90 cells were analyzed using the high-throughput InCell Analyzer 1000 (GE Healthcare) following manufacturer's instructions.

### Western blot analysis

Western blotting was performed by standard procedures. Signals were quantified with LI-COR Odyssey software.

### Senescence-associated β-galactosidase assay

Cells were fixed and stained for SA-βGal by standard protocols. Quantification was performed by two independent observers.

### Quantitative RT–PCR

Primers for TaqMan analysis were from Roche Diagnostics: Samples were normalized to ACTB and RPLP0. Primers for TaqMan analysis were from Roche Diagnostics: ACTB (101125), C/EBPβ (100269), IL-6 (113614), IL-8 (103136), LC3B (144005), and RPLP0 (101144).

### ELISAs and conditioned media

ELISA kits to detect IL-6, IL-8, and TGF-β2 were from Gen-Probe Diaclone. CM was prepared by washing with serum-free DMEM and incubating in serum-free DMEM for 24 h. All ELISA data were normalized to cell number.

### Caspase-1 activity

*Caspase-1 activity* was measured with a caspase-1 fluorometric assay kit (R & D systems) following manufacturer's instructions.

### Flow cytometry

Cell cycle distribution experiments were performed using a FACScan flow cytometer (Becton & Dickinson, San Jose, CA, USA) and analyzed using flowjo software.

### Microarrays

RNA extracts from HCAEC cells were obtained using Trizol® reagent (Life Technologies) and quantified with a NanoDrop 2000 (Thermo Scientific). RNA quality was assessed using a RNA 6000 Nano Kit (Agilent Biotechnologies). Microarrays were prepared using the following kits: Ambion® WT Expression Kit (Life Technologies) and Human GeneChip® Whole Transcript Terminal Labeling Expression Kit (Affymetrix). RNAs were hybridized to GeneChip Human Gene 1.0 ST Arrays. Genes differentially expressed between shNT p7 and p12 cells (senescence genes) were ranked according to their relative expression between shCCM3 and shNT p12 cells and used for gene set enrichment analysis (Subramanian *et al*., 2005) against gene sets from KEGG pathways. The microarray data from this publication have been submitted to the Gene Expression Omnibus Data Repository and assigned the identifier GSE54095.

### Statistical analysis

The statistical significance of all data obtained was assessed by Student's *t*-tests, or ANOVA testing followed by Bonferroni correction where several comparisons were made, using spss software version 12.0.0. All experiments where statistical significance is shown were repeated a minimum of three times.

## Supporting Information

**Fig. S1** Phenotype of late passage CCM3 deficient endothelial cells. **Fig. S2** CCM3 depletion with unrelated shRNAs bypasses OIS in IMR90 cells. **Fig. S3** C/EBPβ but not CCM3 mRNA levels are downregulated in spontaneous meningiomas. **Fig. S4** Transcriptome analysis shows lack of induction of cytokine and lysosome genes in late passage CCM3 depleted cells. **Fig. S5** CCM3 role in autophagy is independent of senescence. **Table S1.** Senescence genes downregulated in the absence of CCM3.
