# Supplementary material for: The cerebral cavernous malformation 3 gene is necessary for senescence induction
Source: Aging Cell. 2015 Feb 5;14(2):274–83. doi: 10.1111/acel.12316 (PMC4364839; doi:10.1111/acel.12316)
Supplement: Supplementary file 1 [file acel0014-0274-sd1.pdf]

Figure S1

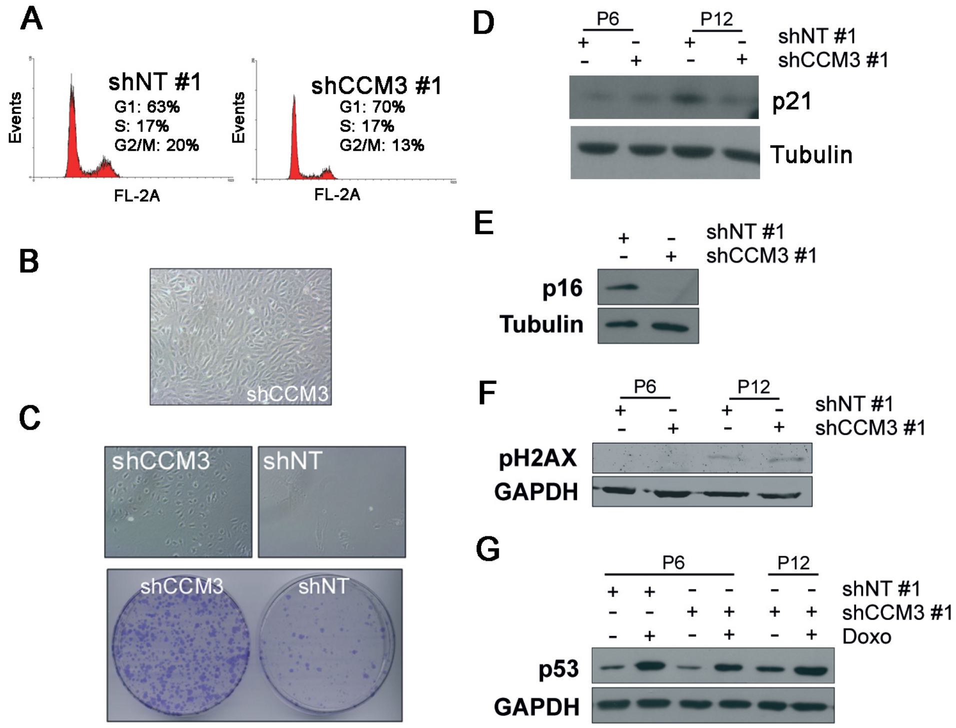

Figure S1. Phenotype of late passage CCM3 deficient endothelial cells (**A**) CCM3 knockdown does not affect cell cycle distribution in early passage cells. Subconfluent shNT and shCCM3#1 HCAEC cells at passage 5 were trypsinized, fixed, and processed for propidium iodine staining. (**B**) CCM3 knockdown cells do not form foci. Passage 18 shCCM3#1 HCAEC cells were allowed to grow to confluence and then kept in complete medium for 10 days and photographed by phase contrast. (**C**) CCM3 knockdown cells are clonogenic. Passage 18 shCCM3#1HCAEC cells were plated at very low density. Ten days later, plates were either photographed by phase contrast (upper panels), or fixed and stained with crystal violet (lower panels). (**D**) Late passage CCM3-deficient cells do not upregulate $p21^{CIP1}$. p21 levels were determined by western blot in shCCM3 and shNT cells at 6 and 12 population doublings. Tubulin is shown as a loading control. (**E**) Late passage CCM3-deficient cells do not upregulate $p16^{ink4a}$. $p16^{ink4a}$ protein levels were determined by western blot in shCCM3 and shNT cells at 12 population doublings. Tubulin is shown as a loading control. (**F**) Late passage CCM3-deficient cells show signs of DNA damage. pH2AX levels were determined by western blot in shCCM3 and shNT cells at 6 and 12 population doublings. GAPDH is shown as a loading control. (**G**) Control (shNT) and shCCM3 HCAEC cells of the indicated population doublings were either left untreated or treated with 1 µM doxorubicin for 24 hours to induce DNA damage. After treatment, p53 levels were assessed by western blot. GAPDH is shown as a loading control.

Figure S2

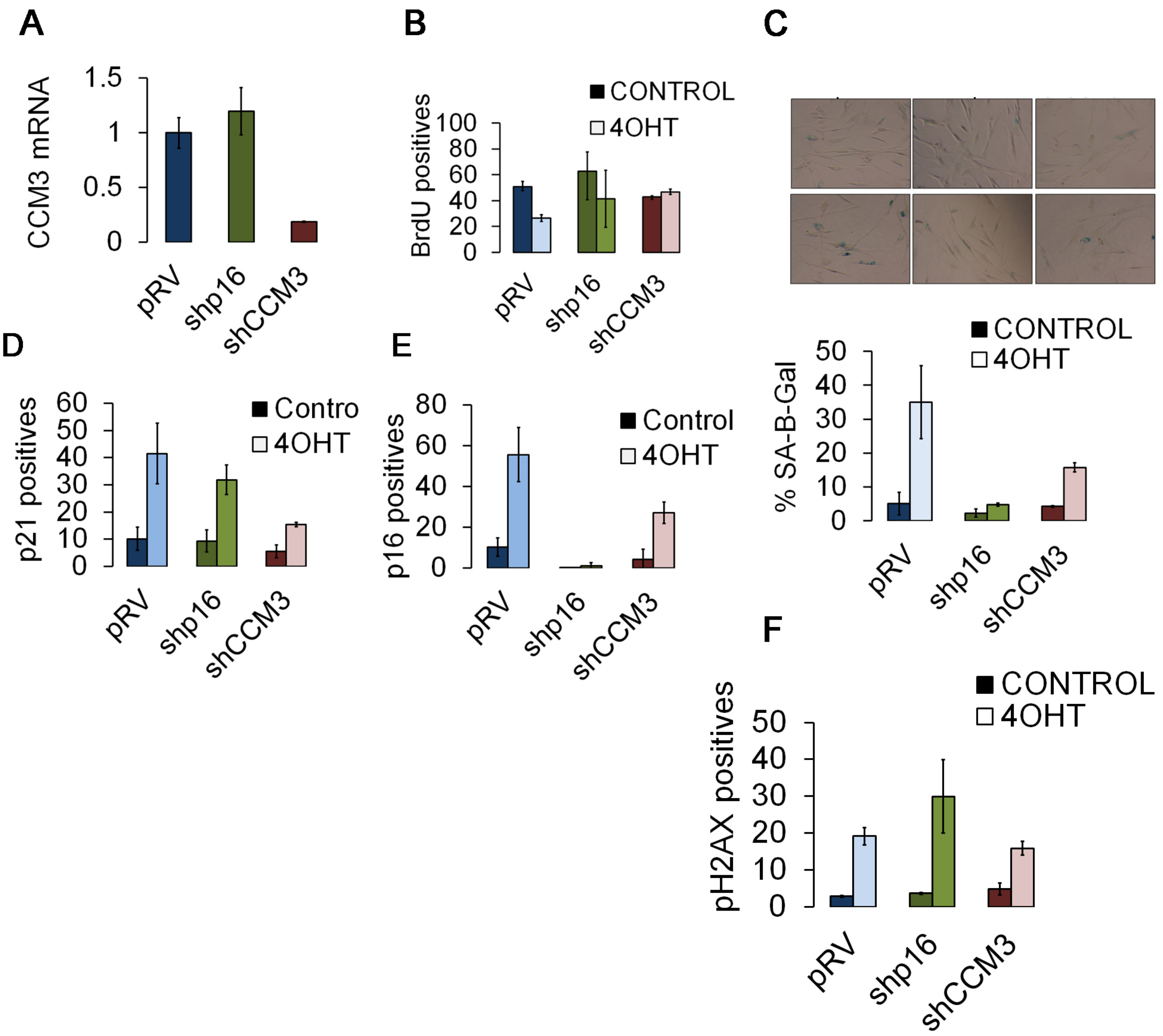

Figure S2. CCM3 depletion with unrelated shRNAs bypasses OIS in IMR90 cells. IMR90 cells expressing a switchable version of Ras (activated upon addition of 4-hydroxitamoxifen, 4-OHT) were retrovirally infected with non-target shRNA (shNT), two different shRNAs against CCM3 or p16 (shp16) and then selected with 0.5 µg/ml puromycin. Cells were also infected with a non-target shRNA (shNT). Shown are the averages of the two CCM3 shRNAs. (**A**) CCM3 mRNA levels were assessed by qRT-PCR in the different populations. (**B**) Percentage of BrdU-positive cells in the different populations untreated or after 5 days of H-Ras activation (4OHT). (**C**) SA-βGal activity in the same cells under the same treatments as in B. Shown are representative photographs and a quantification of SA-βGal positive cells by two independent observers. (**D**) Percentage of $p21^{Kip1}$ positive cells in populations treated as in C. (**E**) Percentage of $p16^{ink4a}$ positive cells in populations treated as in C. (**F**) pH2AX-positive cells in cells treated as in B and C. For D to F, the nuclear intensity average of the staining correlated to the shown percentage of positive cells.

Figure S3

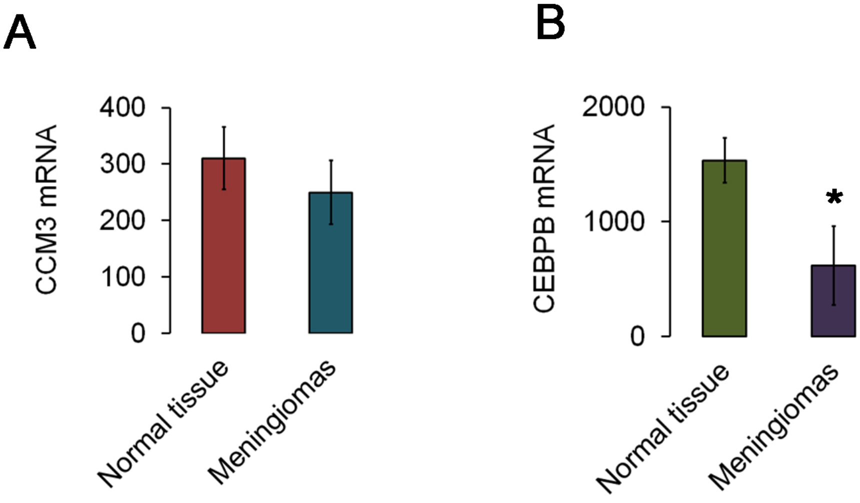

Figure S3. C/EBPβ but not CCM3 mRNA levels are downregulated in spontaneous meningiomas. Shown is the average and standard deviation of CCM3 (**A**) and C/EBPβ (**B**) mRNA levels in normal tissue and meningiomas as analyzed from dataset GSE43290. * $p=3.6 \times 10^{-6}$.

Figure S4

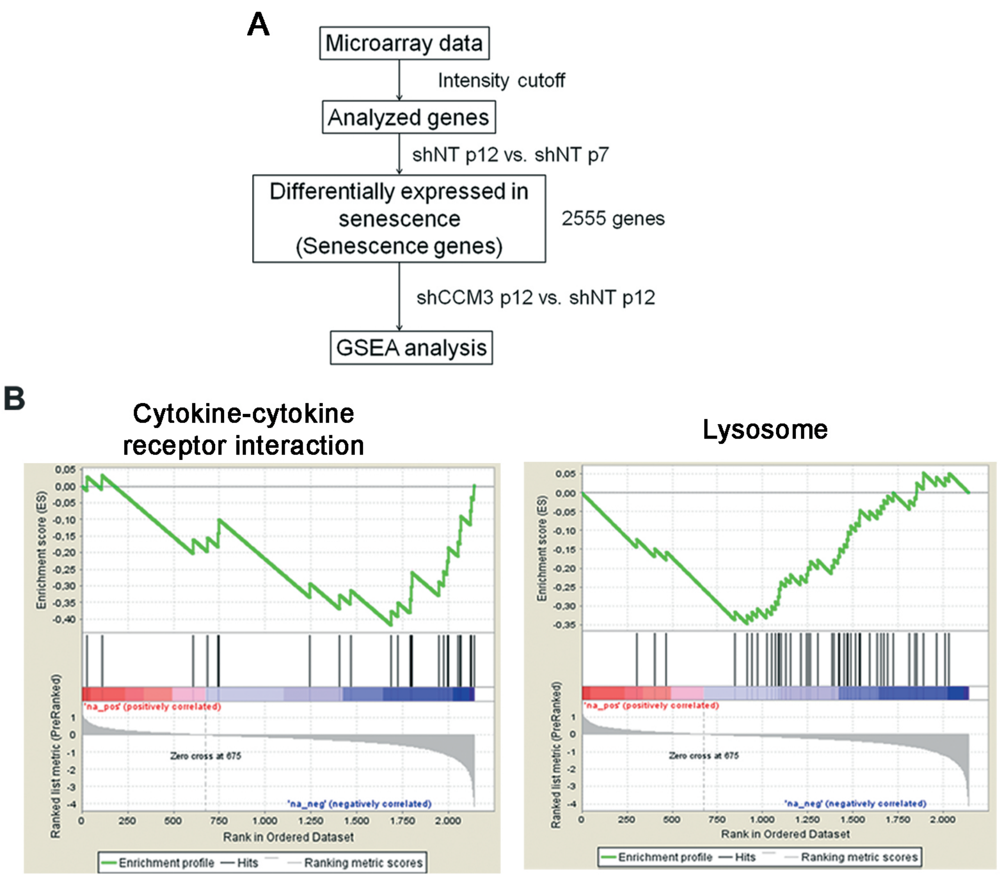

Figure S4: Transcriptome analysis shows lack of inductoin of cytokine and lysosome genes in late passage CCM3 depleted cells. (A) Scheme showing the steps followed in the transcriptomic analysis of senescence in CCM3 deficient cells. **(B)** Gene Set Enrichment Analysis. Normalized Enrichment Score was -2.47 and -2.65 respectively, with a False Discovery Rate of less than 25% in both cases.

Figure S5

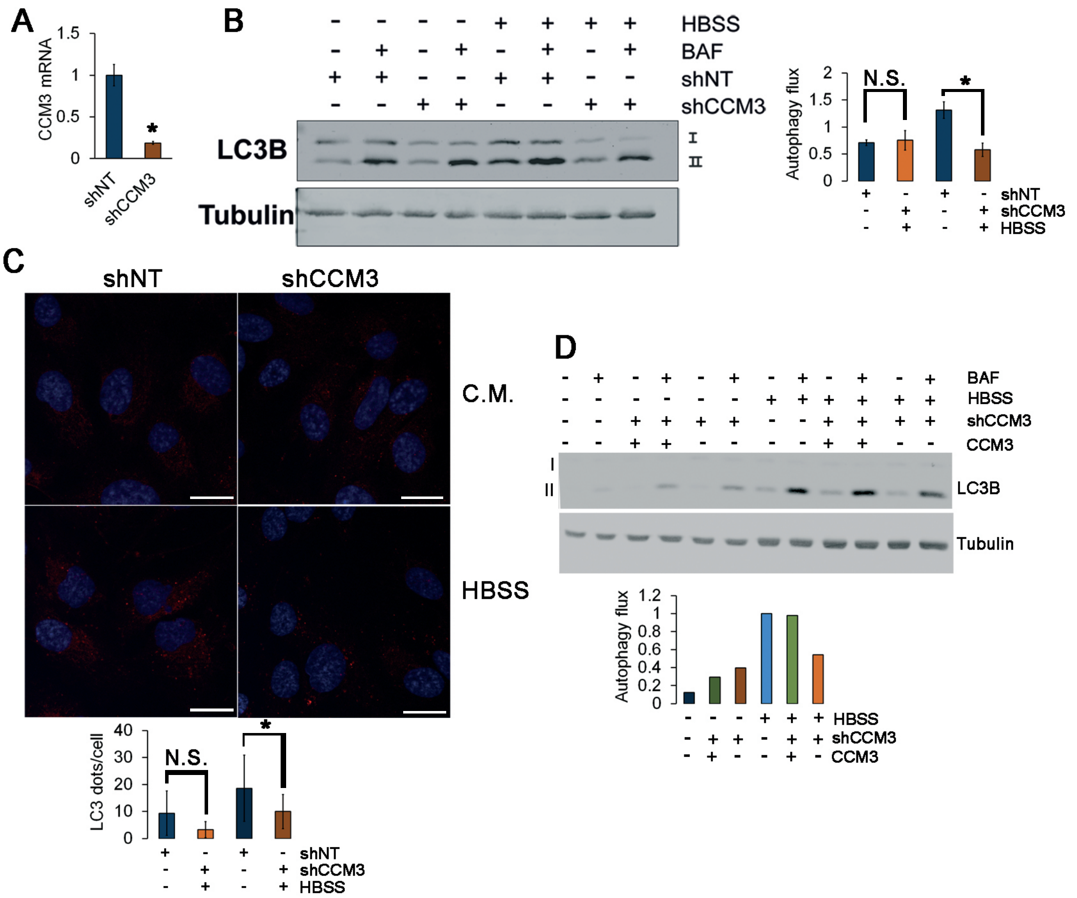

Figure S5. CCM3 role in autophagy is independent from senescence. (**A**) CCM3 mRNA knockdown efficiency in hTERT-RPE1 cells lentivirally infected with an shRNA against CCM3 (shCCM3) compared to non-target shRNA (shNT). n=4, * p=1.5 x 10^{5} vs shNT. (**B**) CCM3 is important for autophagy in immortal cells. hTERT-RPE1 cells were incubated in HBSS or complete medium for 4h. Where indicated, cells were treated with autophagy inhibitor bafilomycin at 100 nM for the last 1h. LC3B-II levels were determined by fluorescent western blot. Tubulin is shown as a loading control. Right panel: autophagic flux under each condition. n=4, N.S. p=1.0, * p=0.018. (**C**) Immunofluorescence of LC3B in hTERT-RPE1 in shNT and shCCM3 hTERT-RPE1 in complete medium or in HBSS for 4 hours. Upper panels: representative micrographs, with bar representing 20 µm. The lower graph is the quantification of LC3B positive punctae under the different treatments. Shown is mean and standard deviation of a minimum of 30 cells. N.S. p=0.093; * p<0.001. (**D**) Rescue of autophagy by CCM3 overexpression. shCCM3 hTERT-RPE1 cells were transduced with pLOC-CCM3 or empty pLOC and autophagic flux was determined as in C.

Supplementary table. Senescence genes downregulated in the absence of CCM3

Gene Symbol	Avg shCCM3 p11	Avg shCCM3 p7	Avg shNT p11	Avg shNT p7	shCCM3 p11/shNT p11	log2(shCCM3 p11/shNT p11)	shNT p11/shNT p7	log2(shNT p11/shNT p7)
CADM3	87.8	59.9	1444.5	190.9	0.061	-4.041	7.567	2.920
TGFB2	83.5	65.3	1261.3	89.2	0.066	-3.918	14.147	3.822
FBLN5	49.4	46.4	526.2	87.9	0.094	-3.414	5.986	2.582
RSPO3	33.7	36.8	345.8	41.9	0.097	-3.361	8.247	3.044
POSTN	425.4	238.6	3296.0	1221.3	0.129	-2.954	2.699	1.432
SULF1	83.9	56.7	609.0	58.6	0.138	-2.860	10.392	3.377
NEXN	27.7	29.5	177.4	38.2	0.156	-2.677	4.644	2.215
HIST1H2BK	126.8	33.9	793.6	79.7	0.160	-2.646	9.960	3.316
PTGS1	149.5	43.7	900.9	418.5	0.166	-2.591	2.153	1.106
PAPSS2	509.2	403.8	2816.5	497.5	0.181	-2.467	5.661	2.501
TPM2	226.2	147.1	1219.8	223.5	0.185	-2.431	5.458	2.448
IL8	187.4	112.8	989.8	392.2	0.189	-2.401	2.524	1.336
IL1RL1	131.5	119.5	618.8	385.9	0.212	-2.235	1.603	0.681
BMPER	22.0	25.6	103.0	27.7	0.214	-2.226	3.719	1.895
FAM38B	65.1	46.8	300.3	117.3	0.217	-2.206	2.560	1.356
TAGLN	983.0	166.5	4397.3	301.9	0.224	-2.161	14.567	3.865
MGP	247.6	84.6	1037.0	139.4	0.239	-2.067	7.440	2.895
CDH2	358.9	327.2	1422.2	523.8	0.252	-1.986	2.715	1.441
FAM38B	147.2	79.9	580.4	186.2	0.254	-1.980	3.117	1.640
KLHL13	37.0	34.3	144.5	61.0	0.256	-1.965	2.368	1.243
GPRC5A	222.0	232.4	859.7	175.8	0.258	-1.953	4.891	2.290
PTPRS	142.0	84.1	535.9	172.9	0.265	-1.916	3.100	1.632
MYL9	648.7	334.2	2445.3	384.9	0.265	-1.914	6.353	2.667
SAT1	439.2	259.2	1646.7	531.3	0.267	-1.907	3.099	1.632
FSTL3	572.0	200.2	2102.4	394.3	0.272	-1.878	5.332	2.415
MLLT11	511.0	210.0	1761.7	647.9	0.290	-1.786	2.719	1.443
PLSCR4	113.4	111.6	365.8	158.8	0.310	-1.690	2.303	1.204
MRC2	120.7	97.3	384.2	165.4	0.314	-1.670	2.322	1.216
LPAR1	12.4	14.5	38.9	13.0	0.320	-1.644	2.988	1.579
BST1	218.8	153.7	683.5	143.2	0.320	-1.643	4.772	2.255
ERRFI1	157.6	166.8	489.9	160.5	0.322	-1.636	3.052	1.610
HSPB8	86.4	90.0	268.3	118.9	0.322	-1.635	2.257	1.175
CDH11	481.1	332.7	1489.1	516.8	0.323	-1.630	2.881	1.527
ANGPTL4	463.3	63.4	1427.7	83.5	0.325	-1.624	17.103	4.096
D4S234E	30.1	29.5	91.5	44.5	0.329	-1.602	2.057	1.040
SLC9A7	50.0	53.2	151.4	89.2	0.330	-1.597	1.697	0.763
INHBA	64.3	40.9	193.5	57.6	0.332	-1.589	3.359	1.748

LASS6	48.6	55.7	146.2	99.2	0.332	-1.589	1.474	0.560
C12orf75	283.5	128.0	848.0	216.0	0.334	-1.581	3.927	1.973
GLIPR1	546.1	513.5	1632.6	646.4	0.335	-1.580	2.526	1.337
PDLIM3	40.3	31.5	120.5	42.2	0.335	-1.579	2.853	1.512
SIPA1L1	61.6	54.0	184.0	73.7	0.335	-1.578	2.497	1.320
KCTD20	65.6	58.4	192.1	88.4	0.342	-1.550	2.174	1.120
HEY2	272.7	124.2	786.4	229.2	0.347	-1.528	3.431	1.778
VCAM1	26.2	23.4	75.1	42.7	0.349	-1.519	1.759	0.815
LOX	388.5	313.8	1080.3	410.3	0.360	-1.475	2.633	1.397
ADAP1	44.9	43.0	122.2	47.7	0.368	-1.444	2.559	1.356
PVR	668.6	448.6	1809.4	466.3	0.370	-1.436	3.880	1.956
MAP1B	596.9	578.2	1607.1	941.7	0.371	-1.429	1.707	0.771
DCBLD2	485.6	439.0	1299.8	753.9	0.374	-1.421	1.724	0.786
ART4	64.5	64.0	171.6	87.8	0.376	-1.411	1.954	0.967
TNFAIP3	49.8	45.0	131.3	44.6	0.379	-1.400	2.943	1.557
FAM101A	408.9	207.3	1078.1	321.9	0.379	-1.399	3.349	1.744
ALCAM	322.9	383.3	841.3	336.7	0.384	-1.381	2.498	1.321
PIP4K2C	34.8	37.0	90.7	48.3	0.384	-1.381	1.877	0.909
ATP6V0A1	156.8	154.5	402.9	165.6	0.389	-1.362	2.434	1.283
PTPRJ	81.4	84.6	208.0	87.8	0.391	-1.354	2.369	1.244
CCDC80	23.9	21.5	61.0	34.0	0.392	-1.353	1.796	0.845
C3orf59	97.6	103.1	246.5	146.9	0.396	-1.337	1.678	0.747
HBEGF	236.1	179.8	594.3	233.9	0.397	-1.332	2.540	1.345
ZNF462	67.4	54.1	168.2	95.9	0.401	-1.320	1.754	0.811
NOTCH2	216.2	205.2	537.5	249.0	0.402	-1.314	2.159	1.110
PPME1	212.4	198.5	527.1	175.0	0.403	-1.311	3.012	1.591
FBN1	221.9	301.4	549.5	227.1	0.404	-1.308	2.419	1.275
SELM	1168.6	252.1	2873.0	235.1	0.407	-1.298	12.222	3.611
CAP2	56.7	44.9	137.6	69.1	0.412	-1.279	1.991	0.993
CPNE7	70.8	62.7	169.6	72.5	0.417	-1.262	2.339	1.226
IGFBP3	113.3	94.7	268.5	83.7	0.422	-1.245	3.208	1.682
ENAH	66.9	47.4	157.9	58.1	0.424	-1.238	2.718	1.443
FLRT2	64.2	34.5	150.5	60.8	0.427	-1.229	2.478	1.309
IGFBP7	2051.5	493.0	4678.2	716.2	0.439	-1.189	6.532	2.708
AEBP1	105.2	86.8	239.5	137.9	0.439	-1.187	1.737	0.796
C5orf30	43.9	42.4	99.8	45.5	0.440	-1.185	2.190	1.131
IL6	86.3	53.9	194.9	78.2	0.443	-1.175	2.492	1.317
CDH13	680.6	453.1	1528.0	418.1	0.445	-1.167	3.654	1.870
PPAPDC1A	168.9	86.6	378.8	166.6	0.446	-1.166	2.273	1.185

ACVR1	71.4	66.9	159.0	89.5	0.449	-1.154	1.778	0.830
DKK3	497.8	172.8	1105.8	443.5	0.450	-1.152	2.493	1.318
VCL	405.3	531.9	886.6	547.2	0.457	-1.129	1.620	0.696
MCFD2	241.9	173.0	522.0	328.6	0.463	-1.109	1.588	0.668
ARMCX6	141.8	104.0	305.3	161.8	0.464	-1.107	1.887	0.916
MYADM	1547.3	673.2	3297.4	898.9	0.469	-1.092	3.668	1.875
CXCL16	91.4	68.9	193.4	111.3	0.472	-1.082	1.737	0.797
PACSIN3	88.9	90.6	184.9	102.8	0.481	-1.057	1.798	0.846
GSTM2	53.2	25.3	110.4	36.8	0.482	-1.053	3.001	1.585
IL13RA1	248.4	202.1	505.9	240.0	0.491	-1.026	2.108	1.076
EXT1	530.7	374.7	1054.1	451.5	0.503	-0.990	2.334	1.223
NAV2	246.4	217.5	487.2	217.7	0.506	-0.983	2.238	1.162
TMTC1	45.7	32.7	89.5	36.6	0.510	-0.971	2.447	1.291
APOBEC3C	243.2	152.7	476.1	182.9	0.511	-0.969	2.603	1.380
ZNF561	39.8	28.5	78.0	34.7	0.511	-0.969	2.248	1.169
CD44	1010.1	612.0	1958.7	821.4	0.516	-0.955	2.385	1.254
C11orf24	228.8	103.3	440.8	106.6	0.519	-0.946	4.135	2.048
SP110	27.7	26.8	53.3	23.5	0.520	-0.945	2.268	1.181
ARMCX6	167.4	138.1	321.3	171.3	0.521	-0.941	1.876	0.908
BCL6	83.4	63.0	158.2	78.0	0.527	-0.924	2.030	1.021
JAG1	313.8	181.6	592.3	377.7	0.530	-0.917	1.568	0.649
LOXL1	126.4	126.0	236.7	145.7	0.534	-0.905	1.624	0.700
DENND5A	201.0	257.1	374.7	274.1	0.537	-0.898	1.367	0.451
COL4A1	197.8	159.1	367.8	306.5	0.538	-0.895	1.200	0.263
WSB2	667.2	294.5	1240.7	416.1	0.538	-0.895	2.982	1.576
SLC6A8	175.8	71.2	326.2	97.3	0.539	-0.892	3.352	1.745
NAV1	549.6	283.9	1012.5	349.9	0.543	-0.882	2.894	1.533
P4HA2	193.7	152.7	353.9	188.1	0.547	-0.870	1.882	0.912
DAB2	1028.0	723.3	1822.8	691.7	0.564	-0.826	2.635	1.398
ANG	36.9	30.9	65.5	46.3	0.564	-0.826	1.415	0.501
SLC6A8	163.6	69.6	289.0	92.9	0.566	-0.821	3.110	1.637
EFEMP2	1041.7	267.8	1835.1	324.3	0.568	-0.817	5.658	2.500
SUGT1P1	25.0	12.5	44.0	19.3	0.568	-0.817	2.282	1.191
TMEM106A	125.5	60.1	220.4	78.4	0.570	-0.812	2.812	1.491
GLA	84.5	64.6	147.9	65.5	0.571	-0.808	2.259	1.176
LGMN	586.3	540.6	1024.5	594.4	0.572	-0.805	1.724	0.786
C11orf41	190.1	131.9	331.0	183.3	0.574	-0.800	1.805	0.852
SNRPN	150.1	115.1	261.4	119.4	0.574	-0.800	2.189	1.130
SELT	1753.1	633.1	3030.1	865.3	0.579	-0.790	3.502	1.808

NDUFC2	535.0	395.4	924.5	477.6	0.579	-0.789	1.936	0.953
FZD8	319.6	239.2	551.9	285.4	0.579	-0.788	1.934	0.951
SLC25A4	94.5	116.4	161.9	118.4	0.584	-0.777	1.367	0.451
PCTP	63.3	33.8	107.3	55.4	0.590	-0.761	1.935	0.952
ACTN1	2397.2	1448.1	4056.0	1423.4	0.591	-0.759	2.849	1.511
TMEM106A	127.5	61.0	215.0	78.6	0.593	-0.753	2.734	1.451
DHRS3	236.9	150.4	395.5	190.8	0.599	-0.739	2.073	1.052
CITED2	591.0	355.2	984.1	304.5	0.601	-0.736	3.232	1.693
CNTNAP1	301.9	221.3	500.7	229.1	0.603	-0.730	2.185	1.128
SPARC	3187.7	1480.6	5285.9	2636.6	0.603	-0.730	2.005	1.003
NEDD9	130.3	91.1	214.9	121.8	0.606	-0.722	1.764	0.819
IPPK	88.1	75.6	145.0	110.6	0.607	-0.720	1.311	0.391
CREB3	680.1	207.2	1119.3	270.3	0.608	-0.719	4.141	2.050
FN1	6592.4	4708.3	10839.8	7441.8	0.608	-0.717	1.457	0.543
SLC25A23	127.1	94.8	208.5	102.5	0.610	-0.714	2.035	1.025
FHL1	449.4	211.0	736.0	179.5	0.611	-0.712	4.100	2.036
RSU1	628.4	419.0	1026.2	448.1	0.612	-0.707	2.290	1.195
RAB32	267.2	197.0	434.8	257.6	0.615	-0.702	1.688	0.755
NNMT	350.8	185.8	569.3	199.2	0.616	-0.699	2.858	1.515
SRPX2	632.2	315.5	1024.5	613.9	0.617	-0.696	1.669	0.739
UBR4	445.1	382.5	719.1	349.8	0.619	-0.692	2.056	1.040
SHC2	100.0	86.8	160.1	94.6	0.625	-0.678	1.692	0.759
TOM1L2	426.7	284.7	680.2	260.2	0.627	-0.673	2.615	1.387
APLP1	114.6	66.2	182.4	71.0	0.628	-0.670	2.570	1.362
SDC3	212.0	87.9	337.3	124.4	0.628	-0.670	2.712	1.439
KIF3C	226.4	156.9	359.1	170.7	0.631	-0.665	2.104	1.073
LOXL2	1765.6	1642.1	2796.0	1734.3	0.631	-0.663	1.612	0.689
REEP5	407.4	294.0	643.5	347.9	0.633	-0.659	1.850	0.887
TFPT	187.7	155.0	296.3	163.7	0.634	-0.658	1.810	0.856
RAB6C	204.1	158.5	320.9	186.1	0.636	-0.653	1.724	0.786
VEGFC	219.2	369.3	344.2	437.6	0.637	-0.651	0.787	-0.346
NDST2	336.6	220.3	527.1	240.8	0.639	-0.647	2.189	1.130
GBF1	368.1	315.0	570.6	332.3	0.645	-0.632	1.717	0.780
ZHX2	72.6	50.8	112.3	62.5	0.647	-0.628	1.797	0.846
MAGED1	869.5	660.7	1340.3	843.2	0.649	-0.624	1.590	0.669
NRG1	117.5	88.9	181.1	121.4	0.649	-0.623	1.492	0.577
IL17RA	259.4	154.2	398.3	170.8	0.651	-0.619	2.332	1.222
GDI1	1532.6	616.3	2349.5	700.1	0.652	-0.616	3.356	1.747
LTBP3	488.2	244.7	748.0	378.6	0.653	-0.616	1.976	0.982

C13orf1	92.8	202.7	140.9	222.2	0.659	-0.601	0.634	-0.658
PACS1	583.1	190.7	882.9	185.8	0.660	-0.599	4.751	2.248
CKAP4	681.5	484.4	1027.8	667.5	0.663	-0.593	1.540	0.623
RCN3	1228.5	1008.4	1835.0	1382.2	0.669	-0.579	1.328	0.409
CLN8	63.6	45.9	94.1	38.9	0.676	-0.565	2.421	1.276
EXT2	371.1	270.4	547.4	271.4	0.678	-0.561	2.017	1.012
GBX2	228.9	221.1	333.1	243.5	0.687	-0.541	1.368	0.452
DDR1	104.8	67.4	151.7	74.1	0.691	-0.534	2.047	1.034
BAG3	308.2	269.2	444.9	315.8	0.693	-0.530	1.409	0.494
PTPN1	382.6	384.6	552.1	406.3	0.693	-0.529	1.359	0.442
TGFBI	495.4	219.7	713.1	382.4	0.695	-0.525	1.865	0.899
SPTAN1	613.8	687.5	883.1	597.3	0.695	-0.525	1.478	0.564
CERCAM	233.7	115.7	334.2	139.1	0.699	-0.516	2.403	1.265
PTPRU	56.2	45.2	80.3	49.0	0.700	-0.515	1.640	0.714
MAP4	278.0	231.9	397.0	241.7	0.700	-0.514	1.643	0.716
TLN1	1188.1	928.7	1684.7	1043.7	0.705	-0.504	1.614	0.691
